# Tissue Paper Softness: A Comparison Between Different Experimental Assessment Approaches

**DOI:** 10.3390/ma18020228

**Published:** 2025-01-07

**Authors:** António de O. Mendes, Joana C. Vieira, Ana M. Carta, Joana M. R. Curto, Maria E. Amaral, Ana P. Costa, Paulo T. Fiadeiro

**Affiliations:** 1 Fiber Materials and Environmental Technologies Research Unit (FibEnTech-UBI), University da Beira Interior, R. Marquês D’Ávila e Bolama, 6201-001 Covilhã, Portugal; 2 Forest and Paper Research Institute (RAIZ), R. José Estevão, Eixo, 3800-783 Aveiro, Portugal

**Keywords:** tissue paper, softness, tissue softness analyzer, subjective evaluation, Kawabata evaluation system, optical system

## Abstract

In this work, four different experimental assessment approaches, namely, the Tissue Softness Analyzer (TSA), a Subjective Evaluation (SUB), the Kawabata Evaluation System (KES), and an Optical System (OPT), were used for the evaluation of softness on a set of 29 different tissue paper products. After processing and the interpretation of the results given by each one of the used methods, a procedure was implemented in the current work to make a comparison between them. The procedure consists in tracking the position of the tissue paper products on a ranking table, regardless of what values were obtained through each one of the four used methods independently. This comparison revealed to be very useful in determining the differences verified between methods allowing to conclude which ones were the least and the most concordant, and, at the same time, enabling us to identify interesting cases of tissue paper products on the set that caught our attention for their distinctive characteristics.

## 1. Introduction

Tissue paper [1,2,3] is an everyday life utility that is everywhere and is widely used by practically everyone. Simply put, at some point, any person will use or be in contact with a product made of tissue, for example, a table napkin, hygienic tissue paper, facial tissue, a paper towel, a kitchen roll, wrapping tissue, and many others [2,4]. Tissue paper has become a multibillion market in size [2,5]. In particular, according to the global data and business intelligence platform Statista [6], the revenue generated in the tissue market worldwide is projected to reach 370.8 billion US dollars in 2025 with an anticipated annual growth of 5.10% in the next five years. In this sense, from a business strategy point of view, the manufacturing companies in the field will continue diversifying their portfolios and improving the quality and the performance of their products in order to keep a high level competitiveness with their rivals, keeping their usual clients satisfied so as to not lose sales, and targeting the arrival of new ones. Of course, when a client is in a store and goes to buy tissue products, he/she encounters dozens or even hundreds of different products from different brands. Thus, with so many options to consider, which product(s) will this person ultimately purchase? This is a question of the most importance and the key for success in the business. Marketing and price are very relevant, and, in fact, there are people that might choose the products they are going to purchase based solely or primarily on the brand or on the price. However, this is not a universal rule, and other aspects are also taken into account even more if the person is uncertain between two or three equivalent products to purchase. It is not very hard to understand that, for instance, when buying a kitchen roll or a paper towel, a product with a good absorption and resistance are paramount, since this kind of product was designed precisely to assist people in cleaning tasks. Thus, the product in question should absorb liquids efficiently and it should be resistant to breaking to not falling apart easily during the task [4,5,7]. If the purchased product delivers in the performed task, the client probably will continue buying it. If not, it may most likely be substituted for a new one, more in line with the demands of the user. Now, if we consider products not designed for this kind of task but, instead, to be used primarily for hygiene or cosmetic purposes, in contact with the skin, one of the most important parameters to consider, if not the most important, is softness [1,2,4,7]. Softness along with strength and absorption properties are influenced by the raw materials and paper process operations [8]. Softness is a very complex feature and relies on many aspects, which is why it has motivated many different studies with very interesting findings that have been published over the years [9,10,11,12,13,14,15,16,17,18,19,20,21,22,23,24,25,26,27,28,29,30,31,32,33]. For instance, a stylus instrument using a modified gramophone cartridge was implemented by Hollmark [9], a mechanical stylus surface analyzer was used by Rust et al. [10], the FRICTORQ equipment was designed by Lima et al. [12], topographic modelling was explored by Rosen et al. [14], a prediction neural model was developed by Rastogi et al. [15], and acoustic emission analysis was used by Kraljevski et al. [30] to address tissue softness. In addition to the presented studies, the works of Hollmark and Ampulski [34] and Pawlak et al. [35] present very comprehensive review studies regarding softness and its measurement that were and are still widely used by different researchers all over the world. On the aforementioned studies, references to the use of subjective panels, the Tissue Softness Analyzer [36], the Kawabata Evaluation System [37,38], and image analysis and processing techniques are very common. What is also very common regarding this topic is the usage of two different methods to assess softness in order to establish a correlation of the results for comparison purposes. The current work aims to accomplish the same goal. However, instead of using only two methods, it explores the four indicated methods to assess softness from a multifaceted perspective on a set of several tissue paper products belonging to the hygiene category. The results obtained in the conducted experiments were then processed and compared to each other, allowing us to organize the tissue products, determine the differences between approaches, identify cases of interest, and, when one or more of the methods differ in their rankings, understand exactly why that happened. The following sections of this work will address the methodologies that were used in our experiments, the principal results that were obtained, and, finally, the main conclusions achieved.

## 2. Materials and Methods

### 2.1. Set of Paper Products

In this work, 29 different paper products of the hygiene tissue category, designated from now on as T01 to T29, were selected to form our set of samples. These products specifically were chosen for our tests due to their market demand, and also because the entire set covers a wide range in terms of softness. As a side note, the initial order of the tissue products in the set are solely based on their arrival at our facilities and is not related with any of their characteristics.

**Figure 1:**
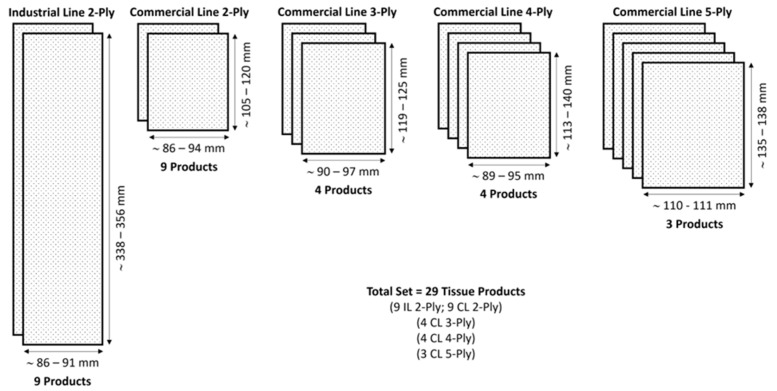
Diagram evidencing the composition of our set of samples, and the typical dimensions of the 2-ply, 3-ply, 4-ply, and 5-ply tissue products.

The majority of the tissue products, 18, to be more precise, are 2-ply papers, of which 9 are from the industrial line, while the other 9 are from the commercial line. Of the remaining 11 products, 4 of them are 3-ply papers, 4 of them are 4-ply papers, and, finally, 3 of them are 5-ply papers, all being from the commercial lines, as depicted in the diagram of Figure 1.

Concerning the dimensions of the products, some points can immediately be highlighted from observation of the diagram. First, the 2-ply products of the industrial line are the most different papers comparatively to all the others, with a width similar to its commercial line, but a height that is approximately 3 times longer. Second, the 5-ply products are considerably wider than all the other products, exceeding 110 mm, while all the others are below 100 mm. Third, in terms of height of the commercial lines, it can be seen that the values range between 105 mm and 140 mm, and the higher height values tend to appear on the products formed with a higher number of plies.

### 2.2. Experimental Methodology

**Figure 2:**
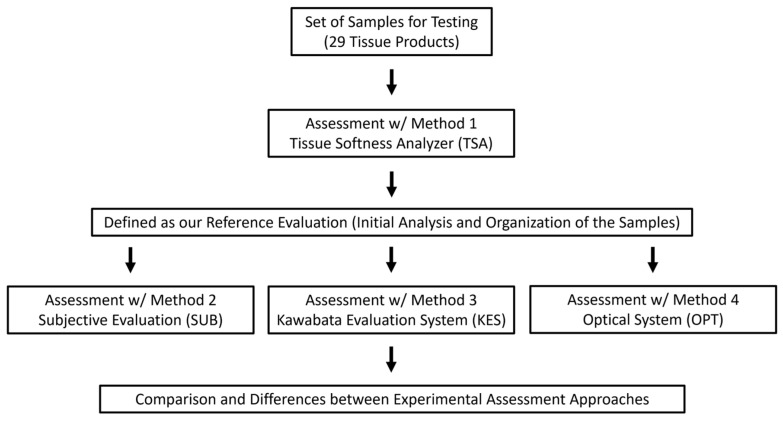
Scheme of the methods implemented experimentally in the current work.

In terms of adopted methodology, 4 different approaches were considered in our experiments, as depicted in the scheme of Figure 2.

The first method, defined as our reference for all the remaining and subsequential work, was the Tissue Softness Analyzer (TSA) [36]. This laboratory equipment simulates the sensation of touch and measures a series of parameters—“real” softness, roughness/smoothness, and stiffness—that are used to estimate a measure of overall softness for the tested tissue papers. This measure is designated handfeel (HF) and is calculated by the TSA equipment through specific algorithms depending on which samples are being tested. In our particular case, the chosen algorithm was the one denominated TPII [36].

For the second implemented method, a Subjective Evaluation (SUB) of all the 29 finished tissue products was carried out in blinded experiments to avoid bias, by 12 different evaluators that have been previously selected to complete this task, depending on their performances in prior tests. In general terms, to each person of the panel was presented a particular sample of the set that he/she had to compare with 5 references in terms of the 3 parameters: bulk, roughness, and flexibility. Then, the evaluators gave a score for each one of the parameters relative to the references. In the end, a global measure of softness for the 29 tissue product samples was calculated based on the 3 tested parameters, considering the average score of all the 12 evaluators in order to improve the accuracy of the method.

The next methodology used by our research team was the Kawabata Evaluation System (KES) [37,38]. This very versatile equipment, composed by different test modules, was used to perform analyses of compression, roughness, and bending, which, similarly for the TSA equipment, allowed us to reach a global value of softness for each one of the 29 tissue products.

The fourth method implemented in the current work was an Optical System (OPT), already used recently in related works, to perform the characterization of different industrial base tissue papers [39], and to study the effect of different embossing patterns [40]. This system was then used to reconstruct the 3D maps of the 29 finished tissue products, and, through those maps, obtain their surface topographies, and other values, related with their roughness, thickness, and bulk, which were also used to calculate an overall measure of softness for the tested products.

All the data acquired with the 4 described methods (TSA, SUB, KES, and OPT) were collected in several experiments carried out at the facilities of the Forest and Paper Research Institute—RAIZ, and University of Beira Interior—UBI.

Finally, in the scheme of Figure 2, one can realize that the Methods section ends with a comparison of the different assessment approaches, which is the core of the whole work. However, it must be pointed out that this comparison of methods was defined from the very beginning with a few considerations to simplify the interpretation of the results. The reason for this was the fact that each method has an enormous amount of data associated with it, especially the KES and the OPT methods. Some of the measured parameters are directly proportional, others are inversely proportional, and some are even represented in different ranges and at different scales, which increases greatly the complexity of the results interpretation, as it is not an easy task to compare them directly.

Having these points in mind, a very simple procedure was taken into account in this work in order to make the comparison between methods straight forward, and easy to understand, since all of them will be expressed in the same way. Basically, the idea is to compare the methods among themselves by simply knowing the position of the tissue products on a ranking table, regardless of what values were obtained through each one of the four used methods independently.

To better understand this idea, let us suppose that only 3 tissue products, A, B, and C, compose our set. By using the TSA equipment on these 3 products, let us say that a high handfeel of 91 is obtained on A, a low handfeel of 50 is obtained on B, and an intermediate handfeel of 68 is obtained on C. For this method, the products should be rearranged as indicated in Table 1.

Now, let us suppose that a second method, for instance, a subjective evaluation, is used on the same 3 tissue products A, B, and C. Through this second method, which uses a different evaluation scale, A is evaluated, let us say, with a 5, as being soft, B is evaluated with a 1, as being rough, and C is evaluated with a 3, as being mild. For this second method, the 3 products should be rearranged as indicated in Table 2.

Finally, the comparison of both methods becomes very easy to perform by simply looking to the position of the rearranged tissue products in Table 1 and Table 2, without the need to compare their initial evaluation values, although, in this particular case, it would be easy due to the simplicity of the presented examples. Specifically, both methods have been shown to be concordant with no deviations in the given ranks. B scored 1 out of 3, C scored 2 out of 3, and A scored 3 out of 3, the best of the three products in terms of softness, for both methods. This is precisely the same procedure that is going to be used in the presentation of the results in the next section of this work for the set of the 29 tested tissue products.

## 3. Results

### 3.1. Comparison Between Methods

The first step regarding the interpretation of the results to our set of samples would be to rearrange them based on the values that were obtained through the reference method, the TSA equipment. Thus, the 29 finished tissue products that compose the set were rearranged from the rougher to the softer, as shown in Table 3.

The first column of Table 3 presents the designation of each one of the tested tissue products, starting with the product T01, and ending with the product T29. The second column indicates their ranks based on the measurements that were obtained with the TSA equipment. The first product T01 shows a TSA rank of 21, meaning that this product had a high value of handfeel, 21 being the highest out of a maximum of 29, and it should go down in the table, toward the softer side, as indicated on the last two columns of Table 3. On the other hand, the last product T29 coincidently shows a TSA rank of 29, meaning that this particular product is in the correct position in the second column of Table 3. It also means that this product, ranked the maximum of 29 in terms of softness, is indeed the softer of the entire set, according to the TSA equipment. The same analysis was conducted for all the remaining tissue products, originating their rearrangement, as can be seen in Table 3.

With this step complete, it becomes easier, from this point forward, to process and interpret the results, because an initial analysis and organization of the products has been carried out, based on their softness characteristics, not being randomly organized anymore. With that said, let us now compare the TSA results with the results obtained through the other three methods (SUB, KES, and OPT), after converting their corresponding measured values from a minimum of 1 to a maximum of 29. Table 4 shows the obtained ranks for each of the assessment methods, having as a reference the TSA method, presented in an ascending order in Table 4 (see column 2).

**Figure 3:**
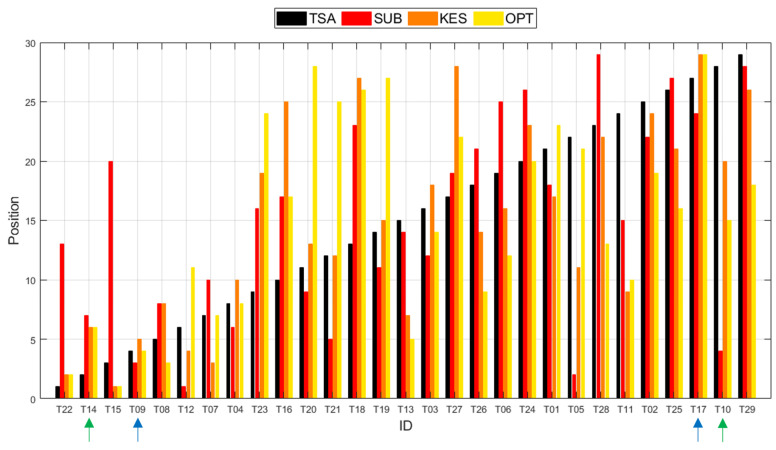
Graphical representation of the positions (ranks) of the 29 tested tissue products by each of the 4 used methods (TSA, SUB, KES, and OPT).

To facilitate the interpretation of the results, the positions (ranks) of the 29 tested tissue products obtained for the four different methods were also represented graphically, in Figure 3. The black bars shown on the graph represent the results using the Tissue Softness Analyzer (TSA), the red bars represent the results obtained in the Subjective Evaluation (SUB), the orange bars represent the results obtained through the Kawabata Evaluation System (KES), and, finally, the yellow bars represent the results that were obtained through the implemented Optical System (OPT).

Through the observation of Figure 3, deviations are seen on the bars across the entire graph, meaning that the methods differ on the given evaluations to the tested tissue products. Ideally, if the methods were indeed 100% concordant, all the bars (black, red, orange, and yellow) should have been represented in the graph in an increasing way, but this was only verified for our reference, the TSA method (black bars). In the graph, it can also be seen that there are cases on which high differences can be detected between two or even more methods, for example, in the cases of the tissue products T15 and T10 (green arrows), meaning that they totally disagree. On the other hand, there are also other cases that can be observed on the graph on which the four methods presented very similar results when compared to each other, such as in the cases of the tissue products T09 and T17 (blue arrows), meaning that there is an agreement between the methods on these cases.

**Figure 4:**
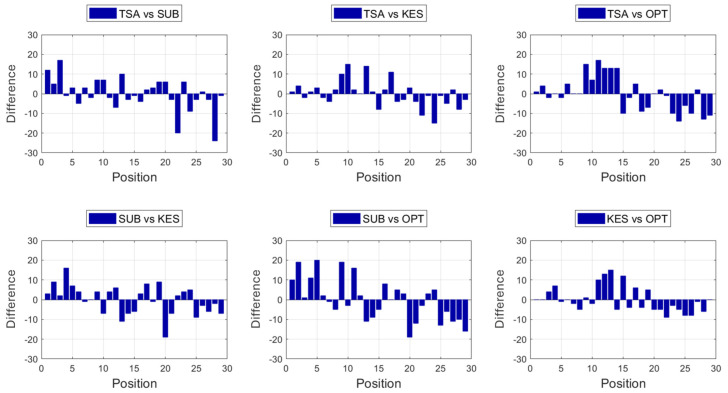
Graphical representation of the differences verified between experimental assessment methods for the 29 tested tissue products.

Continuing the process of comparison between methods, let us now determine the differences verified between the methods two at a time, namely, TSA versus SUB, TSA versus KES, TSA versus OPT, SUB versus KES, SUB versus OPT, and, finally, KES versus OPT. The reference on all the above comparisons corresponds to the first of the two considered methods on each comparison and defines the arrangement of the 29 tissue products from the rougher to the softer. The differences obtained in this process are presented in Table 5, and Figure 4 in graphical form. Table 5 also presents, on the bottom, the average and the standard deviation values that were calculated for the 29 tested tissue products, on the six considered comparisons.

From Table 5, differences can be observed in the comparisons between methods, being positive or negative, and higher or lower, depending on the positions occupied by the tissue products in each one of the used methods. If a particular tissue product goes down on the table comparatively to the reference method, the difference will be positive, and, if it goes up, it will be negative. If the product displaces many positions, the difference will be higher and the corresponding cell will be represented with a darker gray color. On the other hand, if it only displaces a few positions, the difference will be lower and the corresponding cell will be represented with a lighter gray color. All these cases can be seen throughout Table 5. In terms of the biggest differences that were registered, the highest negative value (−24) can be seen on the second position from the end for the comparison TSA versus SUB (darkest cell of the second column of Table 5), whereas the highest positive value (+20) can be seen on the fifth position for the comparison SUB versus OPT (darkest cell of the sixth column of Table 5). The lowest difference (0) appears on several occasions, such as on the twelfth position of the comparison TSA versus KES, on the fourth position of the comparison TSA versus OPT, and so on for the remaining cases. Now, concerning the calculated average of the differences verified between approaches, it is always zero because the negative and the positive differences for each comparison cancel each other out. However, the calculated standard deviations are not zero, and they are revealed to be the lowest for the comparison KES versus OPT, with a value of 6.5. The second lowest standard deviation was registered for the comparison TSA versus KES, with a value of 6.7. This fact can also be verified through Figure 4, by observation of their corresponding graphical representations, which, globally, reveal lower bars. All the other comparison cases are associated with higher standard deviations, being the highest registered for the comparison SUB versus OPT, with a value of 10.7. The corresponding graphical representation of this last comparison, shown in the fifth graph of Figure 4, also suggests the same with bars that, in general, are higher in relation with the other comparison cases. What this means, basically, is that the SUB and OPT methods are the least concordant, whereas the KES and OPT methods are the most concordant of all. Table 6 synthesizes the information of the comparison between both these most concordant methods to be used at the continuation for the analysis of three case studies of interest.

### 3.2. Products Analysis (Rougher, Softer, and Highest Difference)

**Figure 5:**
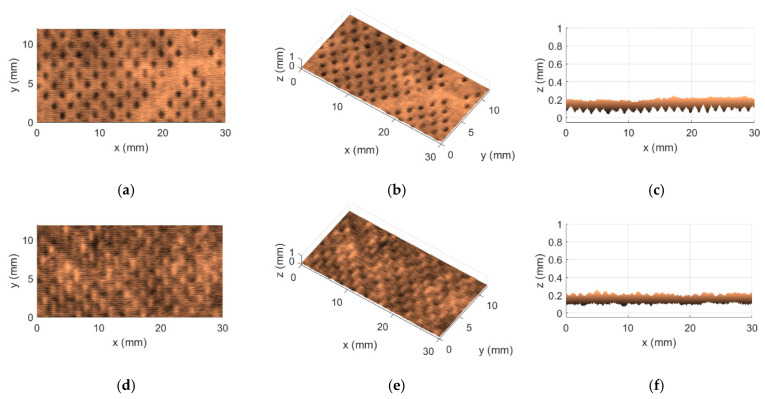
3D map obtained for the tissue product T15: (**a**) top view of front side; (**b**) perspective view of front side; (**c**) lateral view of front side; (**d**) top view of back side; (**e**) perspective view of back side; and (**f**) lateral view of back side.

**Figure 6:**
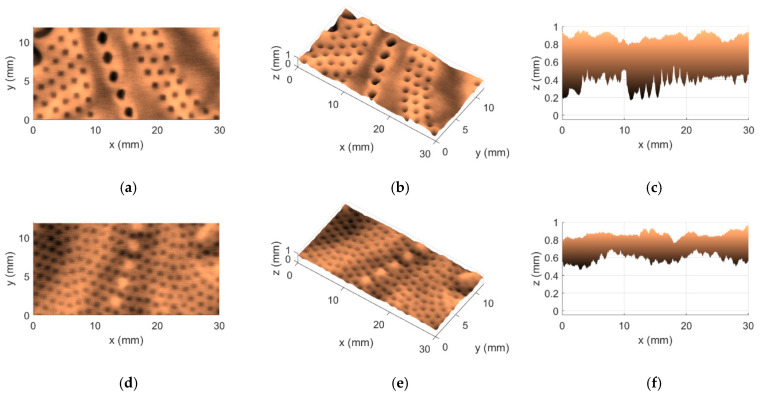
3D map obtained for the tissue product T17: (**a**) top view of front side; (**b**) perspective view of front side; (**c**) lateral view of front side; (**d**) top view of back side; (**e**) perspective view of back side; and (**f**) lateral view of back side.

Regarding the aforementioned two methods, KES and OPT, besides the fact that they were the most concordant of the four that were considered, one other interesting finding caught our attention; in particular, both of them agreed on the selection of the worst and best cases in terms of softness. A difference of zero in the first row and in the last row of the fourth column of Table 6 indicates precisely this point. These cases are associated with the tissue products T15 and T17, respectively. Even more interesting is that these two products are, in fact, very peculiar. They are both very different when compared to each other, being that the product T15 is a very thin 2-ply product of the industrial line, whereas the product T17 is the complete opposite. This last one is a very bulky 5-ply product of the commercial line. Figure 5 and Figure 6 show the 3D maps that were obtained for these two tissue products, T15 and T17, through the use of the implemented Optical System, denoting very well the huge physical differences that exist between both products.

By comparing the views of the 3D maps shown in Figure 5 and Figure 6, some points can immediately be reported: (1) the product T15 is indeed thinner, with values of approximately 0.2 mm measured along the z direction, while the product T17 is a lot thicker, with values of approximately 0.8 mm to 1 mm measured along the z direction; (2) the embossing patterns of both products T15 and T17 are different; and (3) the surfaces of the product T15 seem rougher, revealing a more spiky colormap, while the surfaces of the product T17 appear to be smoother/softer, revealing a more uniform colormap.

**Figure 7:**
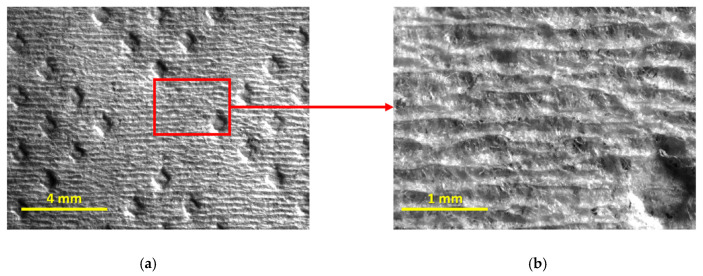
Images of the top surface of the tissue product T15: (**a**) global view; and (**b**) magnified view (4×) of the area contained in the red square.

**Figure 8:**
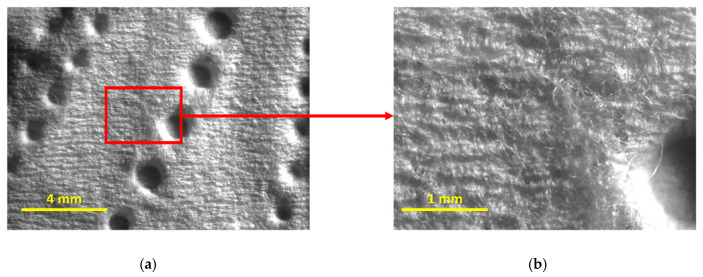
Images of the top surface of the tissue product T17: (**a**) global view; and (**b**) magnified view (4×) of the area contained in the red square.

To help us better analyze these last points, additional images, depicted in Figure 7 and Figure 8, were considered, showing the top surface of both products T15 and T17 in detail.

From Figure 7 and Figure 8, it is indeed possible to verify that the embossing patterns are, in fact, different for the two tissue products, but, more importantly, it is possible to verify that the product T15 is rougher than the product T17. The reason for this is quite simple to understand and is due to the base tissue papers used for the manufacture of both products. From their global views, in Figure 7a and Figure 8a, it is already visible, but, on their magnified views, in Figure 7b and Figure 8b, it becomes even clearer through the observation of their formed patterns of creping lines. The first one, associated with the product T15, is very pronounced and well defined, typically associated with lower handfeel values, and, consequently, lower softness, whereas the second is less pronounced and more diffuse, typically associated with higher values of handfeel, and, consequently, higher softness. This analysis, once again, corroborates the physical differences assessed by the KES and OPT methods that establish these two tissue products as being in extreme positions in terms of softness, specifically, T15 as being the rougher and T17 as being the softer.

To complete the analysis concerning these two products, just one more thing must be done, namely, to verify their positions on the remaining two assessment methods. Table 7 synthesizes the above information for both products.

From Table 7, the TSA method ranked both products very similarly as the KES and the OPT methods, with a value of 3 instead of a 1 for the product T15, and a value of 27 instead of a 29 for the product T17. For both cases, low differences of 2 and −2 were verified, meaning that these three methods are generally in agreement in the evaluation of these two products. Concerning the SUB method, a ranking of 20 and 24 were given to the products. The value of 24 given to the product T17, despite being a bit lower than the values obtained through the other three methods, makes complete sense and continues being in line with the other approaches. The product is still being evaluated as being soft, ranking a 24 out of a maximum of 29. However, for the product T15, the value of 20 that was given in the subjective evaluation differs greatly compared to the other given ranks. From the results obtained through the other three methods, and also by an analysis of the images shown throughout this work, there is nothing that indicates this product as having a softness rank of 20 out of a maximum of 29, clearly appearing to have been overestimated by the subjective panel.

**Figure 9:**
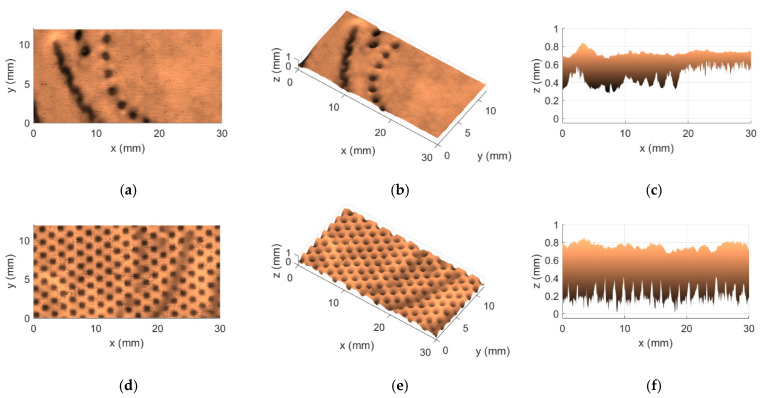
3D map obtained for the tissue product T20: (**a**) top view of front side; (**b**) perspective view of front side; (**c**) lateral view of front side; (**d**) top view of back side; (**e**) perspective view of back side; and (**f**) lateral view of back side.

The last case study considers the product associated with the highest difference between the KES and OPT methods, as shown in Table 6. The 3D map that was obtained through the optical system for this product T20 is presented in Figure 9.

**Figure 10:**
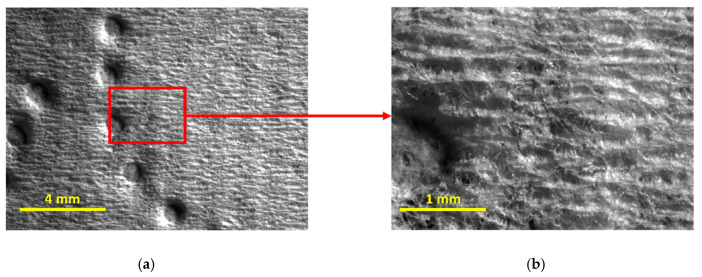
Images of the surface of the front side of the tissue product T20: (**a**) global view; and (**b**) magnified view (4×) of the area contained in the red square.

From the 3D map created with the optical system, the embossing pattern of this product is very different from the previous two, shown in Figure 5 and Figure 6. It can also be seen that this product is also very thick, with values of approximately 0.6 mm to 0.8 mm measured along the z direction. As for the appearance of the surfaces, the colormap suggests that they seem to be not as rough as the product T15, but also not as smooth/soft as the product T17. To help us better analyze this last point, let us consider Figure 10, which shows the top surface of the product T20 in detail.

It can be seen in Figure 10 that, in fact, the surface of the product T20 is not as rough as the product T15, but, on the other hand, it is also not as smooth/soft as the product T17. The creping lines of the base paper are easily distinguishable but more dissimulated, which is revealed to be an intermediate situation between the products T15 and T17. Finally, to complete our analysis concerning this last product, let us verify, in Table 8, the positions (ranks) that were obtained on the four used assessment methods.

From Table 8, this particular product has very similar ranks for the TSA (11), SUB (9), and KES (13), and has a completely different rank for the OPT method (28). This product is a 2-ply commercial product with a high thickness, being a very peculiar case, precisely because of this fact, namely, being very thick but only having two plies on its composition. This is also related to the different rank obtained with the OPT method compared to the other three. By observation of the back side of the product T20, shown in the Figure 9d–f, it can be seen that this surface of the product is filled with an embossing pattern of deep holes. This structure was created on the back side of this product precisely with the purpose of increasing the thickness of the product, which is normally a good thing in terms of the overall softness sensation, due to its increased bulk. However, this created structure also resulted in two other things. The first was an increase in the overall roughness of the paper because the deep holes are easily felt when touched. The second was an increase in the paper’s rigidity. Both things together lead to a product perceived as being less smooth/soft and less flexible, being the most likely reasons why the TSA, SUB and KES methods place this product on the 9–13 ranks of the Table 8. The OPT method, on the other hand, ranked the product T20 as being the second best, with a rank of 28, meaning that it clearly overestimated this product by taking too much into account the thickness of the product, and less into account its other aspects. Considering the obtained results using the four methods, and, by an analysis of the images shown for this last case study, there are no elements that indicate that this product should have such a high softness, meaning that the results obtained with the TSA, SUB, and KES methods make complete sense, clearly suggesting that the OPT method has overestimated it.

## 4. Conclusions

In this work, four different experimental assessment methodologies were used for the evaluation of softness on a set of 29 different tissue paper products. A simple procedure was implemented to compare all methods by simply analyzing the positions of the products on a ranking table, without the need to thoroughly explore the huge amount of data obtained from each one of the used methods.

From the performed comparisons, it was found that the methods that were least concordant were the SUB and the OPT, whereas the methods that were most concordant were the KES and the OPT. For this latter, the best, worst, and highest difference cases were analyzed in detail. For the worst and for the best cases, it was found that the TSA method gave results very similar to the KES and the OPT methods. As for the remaining SUB method, the evaluation of the best case was generally in agreement, with a value perfectly in line with the others, but the worst case was completely off, with a value that was clearly overestimated.

With regard to the case of the product with the highest difference, the TSA, the SUB, and the KES methods were generally in agreement with their corresponding evaluations, but the OPT method was completely off, having performed an overestimation of the product. The reason for this was having taken too much into account the thickness of the tissue product, and too less other of its aspects.

In summary, through this work, it becomes clear the importance of having different assessment approaches available for the evaluation of softness. This happens because softness is clearly a very difficult and complex feature to evaluate, depending on various aspects. By combining all methods together, it is possible to obtain a very complete evaluation of the tissue products, by taking into consideration the best that all have to offer, and better understand what happened on the specific cases on which differences of softness were detected between methods. In this way, it is always possible to confirm the actual grades of softness given to the tissue paper products, and, in case one or more of the methods differ, understand exactly why that happened.

As the focus of future research, the application of this procedure on a vaster set of tissue paper products should be considered in order to further analyze which characteristics of the products are more and less valued by the four tested methodologies.

## Figures and Tables

**Table 1:** Rearrangement of the tissue products A, B, and C based on their TSA results.

Product ID	TSA Handfeel	Rearrangement of the Tissue Products	Product ID	TSA Handfeel	TSA Rank
**A**	High (91)	Rougher	**B**	Low (50)	1
**B**	Low (50)	↓	**C**	Intermediate (68)	2
**C**	Intermediate (68)	Softer	**A**	High (91)	3

**Table 2:** Rearrangement of the tissue products A, B, and C based on their subjective evaluations.

Product ID	Subjective Evaluation	Rearrangement of the Tissue Products	Product ID	Subjective Evaluation	Subjective Rank
**A**	Soft (5)	Rougher	**B**	Rough (1)	1
**B**	Rough (1)	↓	**C**	Mild (3)	2
**C**	Mild (3)	Softer	**A**	Soft (5)	3

**Table 3:** Rearrangement of the 29 tested tissue products based on the TSA method.

Product ID	TSA Rank	Rearrangement of the Tissue Products	Product ID	TSA Rank
**T01**	21		**T22**	1
**T02**	25		**T14**	2
**T03**	16		**T15**	3
**T04**	8		**T09**	4
**T05**	22		**T08**	5
**T06**	19		**T12**	6
**T07**	7		**T07**	7
**T08**	5		**T04**	8
**T09**	4		**T23**	9
**T10**	28		**T16**	10
**T11**	24		**T20**	11
**T12**	6		**T21**	12
**T13**	15		**T18**	13
**T14**	2	Rougher	**T19**	14
**T15**	3	↓	**T13**	15
**T16**	10	Softer	**T03**	16
**T17**	27		**T27**	17
**T18**	13		**T26**	18
**T19**	14		**T06**	19
**T20**	11		**T24**	20
**T21**	12		**T01**	21
**T22**	1		**T05**	22
**T23**	9		**T28**	23
**T24**	20		**T11**	24
**T25**	26		**T02**	25
**T26**	18		**T25**	26
**T27**	17		**T17**	27
**T28**	23		**T10**	28
**T29**	29		**T29**	29

**Table 4:** Corresponding positions (ranks) of the 29 tested tissue products obtained for the 4 considered assessment methods.

	Ranks			
Product ID	TSA	SUB	KES	OPT
**T22**	1	13	2	2
**T14**	2	7	6	6
**T15**	3	20	1	1
**T09**	4	3	5	4
**T08**	5	8	8	3
**T12**	6	1	4	11
**T07**	7	10	3	7
**T04**	8	6	10	8
**T23**	9	16	19	24
**T16**	10	17	25	17
**T20**	11	9	13	28
**T21**	12	5	12	25
**T18**	13	23	27	26
**T19**	14	11	15	27
**T13**	15	14	7	5
**T03**	16	12	18	14
**T27**	17	19	28	22
**T26**	18	21	14	9
**T06**	19	25	16	12
**T24**	20	26	23	20
**T01**	21	18	17	23
**T05**	22	2	11	21
**T28**	23	29	22	13
**T11**	24	15	9	10
**T02**	25	22	24	19
**T25**	26	27	21	16
**T17**	27	24	29	29
**T10**	28	4	20	15
**T29**	29	28	26	18

**Table 5:** Rank differences obtained in the comparison between assessment methods for the 29 tested tissue products.

	Rank Differences Between Approaches					
Product ID	TSA vs. SUB	TSA vs. KES	TSA vs. OPT	SUB vs. KES	SUB vs. OPT	KES vs. OPT
**T22**	+12	+1	+1	+3	+10	**0**
**T14**	+5	+4	+4	+9	+19	**0**
**T15**	+17	−2	−2	+2	+1	+4
**T09**	−1	+1	**0**	+16	+11	+7
**T08**	+3	+3	−2	+7	**+20**	−1
**T12**	−5	−2	+5	+4	+2	**0**
**T07**	+3	−4	**0**	−1	−1	−2
**T04**	−2	+2	**0**	**0**	−5	−5
**T23**	+7	+10	+15	+4	+19	+1
**T16**	+7	+15	+7	−7	−3	−2
**T20**	−2	+2	+17	+4	+16	+10
**T21**	−7	**0**	+13	+6	+2	+13
**T18**	+10	+14	+13	−11	−11	+15
**T19**	−3	+1	+13	−7	−9	−5
**T13**	−1	−8	−10	−6	−5	+12
**T03**	−4	+2	−2	+3	+8	−4
**T27**	+2	+11	+5	+8	**0**	+6
**T26**	+3	−4	−9	−1	+5	−4
**T06**	+6	−3	−7	+9	+3	+5
**T24**	+6	+3	**0**	−19	−19	−5
**T01**	−3	−4	+2	−7	−12	−5
**T05**	−20	−11	−1	+2	−3	−9
**T28**	+6	−1	−10	+4	+3	−3
**T11**	−9	−15	−14	+5	+5	−5
**T02**	−3	−1	−6	−9	−13	−8
**T25**	+1	−5	−10	−3	−6	−8
**T17**	−3	+2	+2	−6	−11	−1
**T10**	**−24**	−8	−13	−2	−10	−6
**T29**	−1	−3	−11	−7	−16	**0**
**Average**	0.0	0.0	0.0	0.0	0.0	0.0
**Standard Deviation**	8.4	6.7	8.7	7.4	10.7	6.5

**Table 6:** Main results of the comparison between the KES and OPT methods.

Product ID (Order Based on KES)	KES Rank	OPT Rank	Differences (KES vs. OPT)
**T15**	1	1	0
**T22**	2	2	0
**T07**	3	7	+4
**T12**	4	11	+7
**T09**	5	4	−1
**T14**	6	6	0
**T13**	7	5	−2
**T08**	8	3	−5
**T11**	9	10	+1
**T04**	10	8	−2
**T05**	11	21	+10
**T21**	12	25	+13
**T20**	13	28	+15
**T26**	14	9	−5
**T19**	15	27	+12
**T06**	16	12	−4
**T01**	17	23	+6
**T03**	18	14	−4
**T23**	19	24	+5
**T10**	20	15	−5
**T25**	21	16	−5
**T28**	22	13	−9
**T24**	23	20	−3
**T02**	24	19	−5
**T16**	25	17	−8
**T29**	26	18	−8
**T18**	27	26	−1
**T27**	28	22	−6
**T17**	29	29	0

**Table 7:** Basic characteristics and corresponding positions (ranks) of the products T15 and T17 obtained for the 4 considered assessment methods.

Product ID	Line	Thickness	Number of Plies	TSA Rank	SUB Rank	KES Rank	OPT Rank
**T15**	Industrial	Low	2	3	20	1	1
**T17**	Commercial	High	5	27	24	29	29

**Table 8:** Basic characteristics and corresponding positions (ranks) of the product T20 obtained for the 4 considered assessment methods.

Product ID	Line	Thickness	Number of Plies	TSA Rank	SUB Rank	KES Rank	OPT Rank
**T20**	Commercial	High	2	11	9	13	28

## Data Availability

The original contributions presented in this study are included in the article material. Further inquiries can be directed to the corresponding author.
